# Adlay (*Coix lachryma-jobi* L. var. *ma-yuen* Stapf.) Hull Extract and Active Compounds Inhibit Proliferation of Primary Human Leiomyoma Cells and Protect against Sexual Hormone-Induced Mice Smooth Muscle Hyperproliferation

**DOI:** 10.3390/molecules24081556

**Published:** 2019-04-19

**Authors:** Po-Han Lin, Chun-Kuang Shih, Yu-Ting Yen, Wenchang Chiang, Shih-Min Hsia

**Affiliations:** 1 School of Nutrition and Health Sciences, College of Nutrition, Taipei Medical University, Taipei 11031, Taiwan; phlin@tmu.edu.tw (P.-H.L.); ckshih@tmu.edu.tw (C.-K.S.); 2 Institute of Food Science and Technology, National Taiwan University, Taipei 10617, Taiwan; eillenlily@hotmail.com (Y.-T.Y.); chiang@ntu.edu.tw (W.C.); 3 Graduate Institute of Metabolism and Obesity Sciences, College of Nutrition, Taipei Medical University, Taipei 11031, Taiwan; 4 School of Food Safety, College of Nutrition, Taipei Medical University, Taipei 11031, Taiwan; 5 Nutrition Research Center, Taipei Medical University Hospital, Taipei 11031, Taiwan

**Keywords:** uterine leiomyomas, fibroids, adlay hull, stigmasterol

## Abstract

Uterine leiomyomas, also known as fibroids, are benign neoplasms of the uterus and have a high incidence rate in women of reproductive age. Hysterectomy or myomectomy is the initial treatment, but fibroids will recur if the patient is still exposed to similar risk factors. Therefore, developing new therapeutic strategies are urgently necessary. In this study, the anti-proliferation effects of each fraction of adlay seeds were evaluated in uterine leiomyomas, and we identified the potential phytochemical compounds. We found that the ethyl acetate fraction of adlay hull (AHE-ea) appeared to be highly efficient in the anti-proliferation of rat uterine leiomyoma ELT3 cells and primary human uterine leiomyoma (hUL) cells. The proliferation of primary human normal uterine smooth muscle (UtSMC) and normal uterine myometrial (hUM) cells were also suppressed by AHE-ea. Two phytosterols, stigmasterol and β-sitosterol, were identified from AHE-ea fraction. Mice treated with AHE-ea and stigmasterol alone demonstrated reduced diethylstilbestrol/medroxyprogesterone 17-acetate (DES/MPA)-induced uterine myometrial hyperplasia, which is the critical step for the development of leiomyoma. Taken together, our results suggest that the AHE-ea fraction could be considered as a natural plant-based medicine in the prevention or treatment of uterine leiomyoma growth.

## 1. Introduction

Uterine leiomyomas, also known as fibroids, are a common female reproductive disorder. They are benign neoplasms originating from myometrial smooth muscle cells of the uterus and often occur in women of premenopausal age [1,2,3]. Uterine leiomyomas can be asymptomatic until an enlarged uterus or a mass is palpated during a pelvic examination or after a patient reports heavy menstrual bleeding [4,5]. Ultrasonography-screening examination is commonly used to confirm the status and location of uterine leiomyoma. The symptoms commonly associated with uterine leiomyomas are major gynecological problems. Painful menstruation, irregular pelvic pain, and pressure, as well as heavy menstrual bleeding are the common symptoms in women with uterine leiomyomas [1,6,7]. The current and well-recognized therapeutic strategy for uterine leiomyoma is hysterectomy or myomectomy [2,8]; however, recurrence from underlying risk factors is still possible [9]. Therefore, there is an urgent need to develop new pharmacotherapies to tackle uterine leiomyoma.

Plant-based medicines have long been used in the prevention or treatment of the diseases [10,11,12]. Adlay (*Coxi lachrtma-jobi* L. var. *ma-yuen* Stapf.) has high nutritional value. It is widely cultivated in Asia and has long been consumed as both food in cuisine and used as a traditional Chinese medicine. Adlay has been reported to have anti-inflammatory and antioxidant properties and has been used in the treatment of edema, rheumatism, and neuralgia [13,14,15,16]. Many components of adlay have been identified as having biological functions; adlay-related proteins can improve type II diabetes [17] and the oil fraction of adlay seeds has displayed an effect on the reduction of blood lipids in hyperlipidemic rats [18]. 

The antitumor effect of adlay has been investigated in preclinical studies. The efficacy of adlay extracts on the suppression of tumor proliferation, including in lung, breast, colon, and leukemia cancer cells, has been determined [19,20,21,22]. An oil extracted from adlay seeds, named Kanglaite injection (KLTi), has been shown to inhibit colon cancer metastasis via suppressing NF-κB-induced epithelial-mesenchymal transition [23]. KLTi enhances Taxol therapeutic efficacy on the suppression of colon cancer growth in vitro and in vivo [24]. KLTi has been approved by the Food and Drug Administration (FDA) in the United States for Phase II clinical trials in a combination of KLTi and chemotherapy for enhancing therapeutic efficacy in non-small-cell lung, gastric, and pancreatic cancers [25,26,27].

Regarding the effect of adlay on the female reproductive system, the Traditional Chinese Medicine Classic suggests that women have to avoid a diet of adlay during pregnancy [28]. This argument has been confirmed by an animal study, which reported that treated rats with water extract of adlay seed demonstrated significantly enhanced spontaneous uterine contractions via the induction of PKCα and ERK1/2 activity as well as cyclooxygenase-2 (COX-2) protein expression [29]. We previously reported that treatment of rat uterus with the extracts of adlay hull reduce the PGF2α-induced uterine contraction and intrauterine pressure [30]. We also found that combined extracts of adlay testa and doxorubicin, a widely used medicine for treating several cancer cell types, can decrease multidrug resistance and increase the synergistic effect on anti-proliferation in human uterine sarcoma cells [31]. These results suggest adlay has the potential to improve pelvic pain and the treatment of uterine tumor growth. Adlay is the major ingredient in Gong Zheng Tang, one of the traditional Chinese herbal medicine references, and it has been applied in a clinical trial for patients with uterine leiomyoma. In these 136 cases, 72 (53%) were cured and another 37 cases (26%) were significantly improved [32]. However, until now the efficiency of adlay extracts and its active compounds on suppressing the growth of uterine leiomyoma is still unclear.

In the present study, the adlay seeds were separated into four parts: hull, testa, bran, and polished adlay. The ethanolic extracts from these four parts were fractioned using the following solvents: n-hexane and ethyl acetate. We investigated the effect of adlay extracts on the growth of uterine leiomyoma both in vitro and in vivo. Our results demonstrated that the ethyl acetate fraction of adlay hull ethanolic extract exhibits high efficiency in anti-proliferation of uterine leiomyoma cells and the reduction of female sexual hormone-induced uterine hyperplasia in vivo. These findings highlight a novel role of adlay in the anti-proliferation of uterine leiomyoma.

## 2. Results

### 2.1. Effects of the Ethanolic Extracts from Four Parts of Adlay Seed on ELT3 Cell Viability

**Figure 1:**
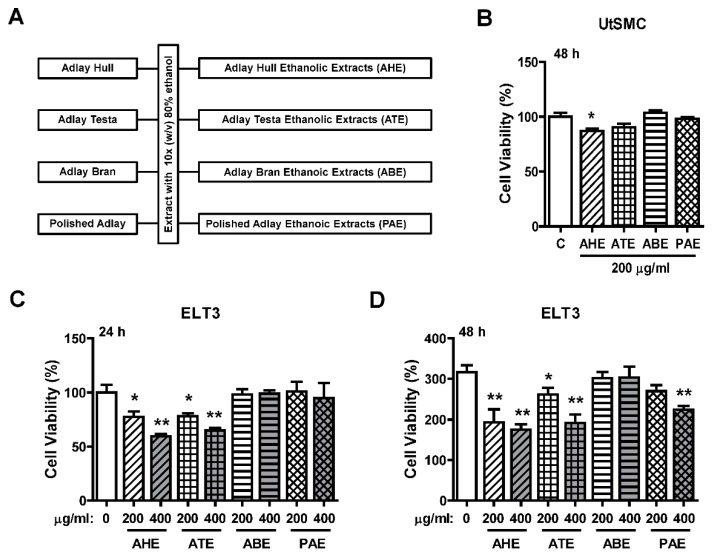
Effects of adlay seed ethanolic extract on the viability of UtSMC and ELT3 cells. (**A**) The ethanolic extraction process of the different parts of the adlay seed. (**B**) Human UtSMC (2500 cells/well) were seeded in 96-well plates. UtSMC cells were treated with different parts of adlay seed ethanolic extracts (200 μg/mL) for 48 h. ELT3 cells (2500 cells/well) were seeded in 96-well plates. ELT3 cells were treated with different parts of the seeds extracted with ethanolic extracts at 200 and 400 μg/mL (**C**) for 24 h and (**D**) 48 h. At the end of incubation, the inhibitory capacity of these ethanolic extracts was evaluated using the MTT assay. Data represent means ± SEM (*n* = 4–6). * *p* < 0.05, ** *p* < 0.01 compared with the control group.

The ethanolic extraction process of four parts of adlay (AHE, ATE, ABE, and PAE) is shown in Figure 1A. To evaluate whether adlay has the potential to suppress leiomyoma growth, rat uterine leiomyoma ELT3 cells and primary human normal uterine myometrial cells (UtSMC) were treated with these four parts (AHE, ATE, ABE, and PAE). The results showed that the cell viability was slightly inhibited by ATE treatment at 200 μg/mL for 48 h. The other three parts of adlay did not affect growth (Figure 1B). In contrast, after treatment for 24 h, AHE and ATE appeared to have an inhibitory effect on ELT3 cell growth at 200 and 400 μg/mL (Figure 1C). After 48 h, this inhibitory effect was significantly observed by treatment of ELT3 with AHE and ATE at 200 and 400 μg/mL, respectively (Figure 1D). In addition, treated ELT3 with PAE at 400 μg/mL for 48 h also inhibited cell viability (Figure 1D). These results suggested that AHE and ATE could be the parts of adlay that have the ability to suppress leiomyoma growth.

### 2.2. Effects of Each Fraction of AHE and ATE on ELT3 Cell Viability

**Figure 2:**
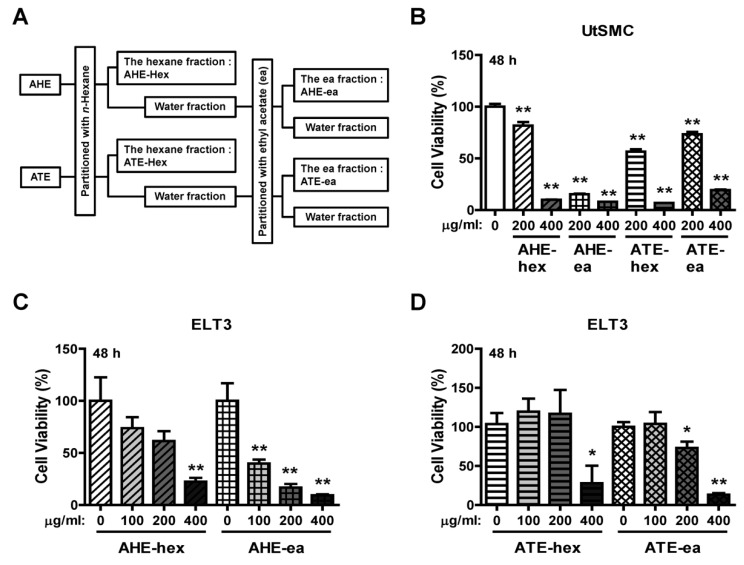
Effects of hexane and ethyl acetate fractions from AHE and ATE on the viability of UtSMC and ELT3 cells. (**A**) The hexane and ethyl acetate fractions from AHE and ATE. (**B**) Human UtSMC (2500 cells/well) were seeded in 96-well plates. UtSMC cells were treated with the hexane and ethyl acetate fractions of AHE and ATE (200 and 400 μg/mL) for 48 h. ELT3 cells (2500 cells/well) were seeded in 96-well plates. ELT3 cells were treated with the hexane and ethyl acetate fractions of AHE and ATE at concentrations of 100, 200 and 400 μg/mL for (**C**) 24 h and (**D**) 48 h. At the end of incubation, the inhibitory capacity of these extracts was evaluated using the MTT assay. Data represent means ± SEM (*n* = 6). * *p* < 0.05, ** *p* < 0.01 compared with the control group.

AHE and ATE were further partitioned by mixing with n-hexane and ethyl acetate; the extraction process is shown in Figure 2A. The yield of each sub-fraction is provided in Table 1. UtSMC and ELT3 cells were treated with these fractions to evaluate their anti-proliferation efficiency. The results showed that AHE-hex, ATE-hex and ATE-ea at 200 μg/mL had an approximately 20–40% suppressive efficiency on UtSMC cell growth after treatment for 48 h. Treatment of UtSMC with the AHE-ea fraction at 200 μg/mL for 48 h exhibited an 80–90% suppressive efficiency on cell growth. An 80–90% of suppressive efficiency was also observed when cells were treated with these four fractions at 400 μg/mL (Figure 2B). A similar pattern was found in ELT3 cells. After treatment with AHE-hex, ATE-hex and ATE-ea for 48 h, their anti-proliferation efficiency significantly appeared at the dosage of 400 μg/mL (Figure 2C,D). However, ELT3 cells treated with the AHE-ea fraction at 100 μg/mL suppressed cell growth by approximately 50–60% and suppression higher than 70% was observed with dosages of 200 and 400 μg/mL (Figure 2C).

**Figure 3:**
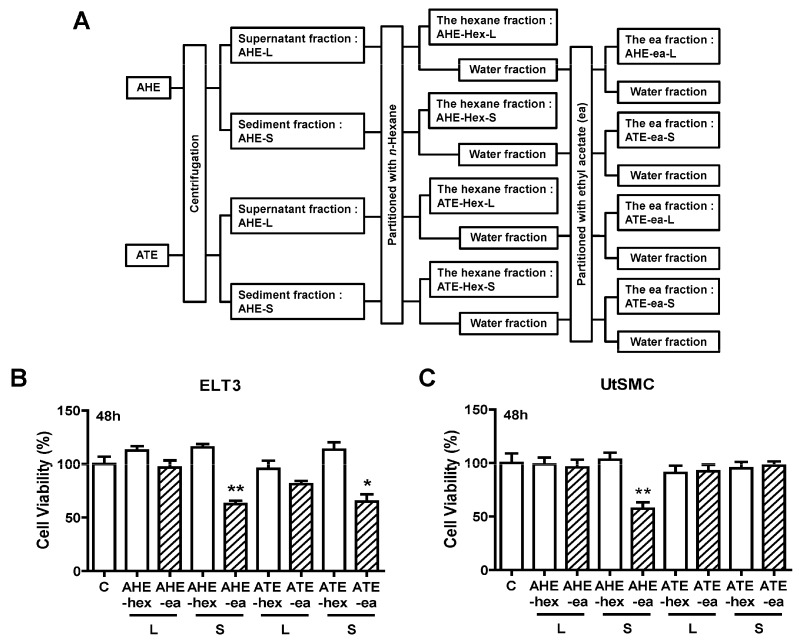
Effects of hexane and ethyl acetate fractions from the supernatant and sediment of AHE and ATE on the viability of UtSMC and ELT3 cells. (**A**) The hexane and ethyl acetate fractions from the supernatant and sediment of AHE and ATE. (**B**) ELT3 and (**C**) UtSMC cells (2500 cells/well) were treated with the hexane and ethyl acetate fractions of AHE and ATE at a concentration of 100 μg/mL for 48 h. At the end of incubation, the inhibitory capacity of these extracts was evaluated using the MTT assay. Data represent means ± SEM (*n* = 6). * *p* < 0.05, ** *p* < 0.01 compared with the control group. C, control. L, supernatant phase. S, sediment phase.

Other partition process from AHE and ATE fractions were performed in this study. Before partition, the AHE and ATE fractions in the supernatant were separated from the sediment fractions by centrifugation. Subsequently, each fraction was mixed with n-hexane and ethyl acetate as shown in Figure 3A. The yield of each sub-fraction is listed in Table 2. The potential phase on anti-proliferation was also evaluated from these fractions. The results showed that only AHE-ea-S and ATE-ea-S (100 μg/mL) treatment for 48 h reduced ELT3 cell growth by approximately 50%. The other fractions did not affect ELT3 cell growth (Figure 3B). In UtSMC cells, the AHE-ea-S (100 μg/mL) was the only fraction that reduced cell growth after treatment for 48 h (Figure 3C).

### 2.3. Effects of Each Fraction of AHE and ATE on Primary Human Uterine Leiomyoma (hUL) Cells

**Figure 4:**
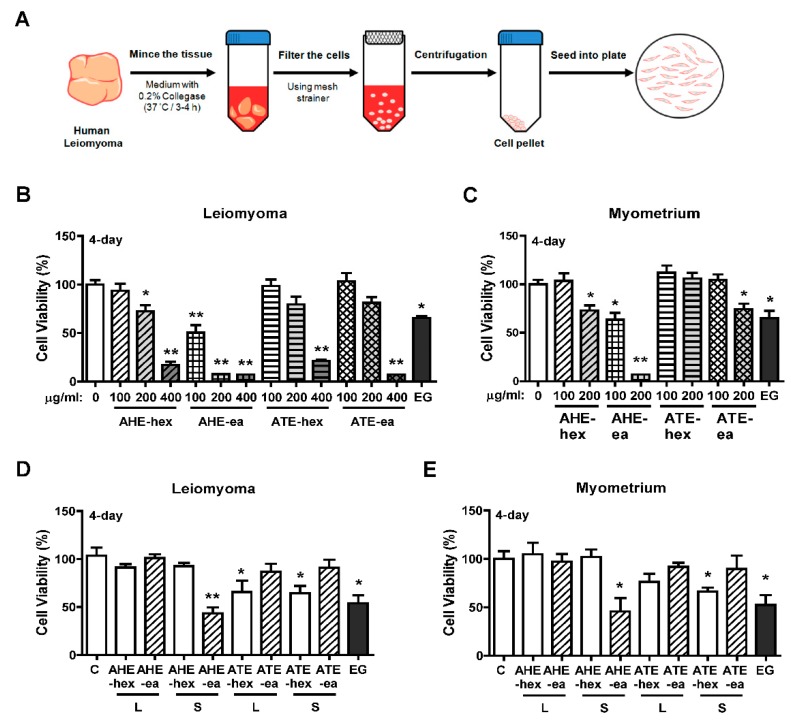
Effects of hexane and ethyl acetate fractions from AHE and ATE on the viability of human primary uterine leiomyoma (hUL) and myometrial (hUM) cells. (**A**) Diagram of the process of human primary uterine leiomyoma isolation; a similar process was performed to isolate human primary uterine myometrial cells. Both hUL and hLM cells were seeded at 2000 cells per each well of 96-well plates. Serial concentrations of the hexane and ethyl acetate fractions from AHE and ATE were used for treatment of (**B**) hUL and (**C**) hUM cells for 4 days. Cell viability was detected using an MTT assay. (**D**) hUL and (**E**) hUM cells were treated with hexane and ethyl acetate fractions from the supernatant and sediment of AHE and ATE for 4 days, and then the cell viability was detected using the MTT assay. Data represent means ± SEM (*n* = 6). * *p* < 0.05, ** *p* < 0.01 compared with the control group. C, control. L, supernatant phase. S, sediment phase. EG, epigallocatechin gallate (EGCG; 100 μM), as a positive control.

To evaluate whether each fraction of AHE and ATE has a suppressive effect on human uterine leiomyoma growth, primary human uterine leiomyoma (hUL) cells and normal uterine myometrial (hUM) cells were purified from uterine leiomyoma and normal uterine myometrial tissues, respectively. The isolation process is shown in Figure 4A. We found that the growth of hUL cells was suppressed by treatment with AHE-hex at 200 and 400 μg/mL and AHE-ea at 100, 200, and 400 μg/mL for 4 days. hUL cells treated with ATE-hex and ATE-ea fractions for 4 days demonstrated reduced cell growth when the dosage was 400 μg/mL (Figure 4B). Treatment of epigallocatechin gallate (EGCG) was reported to suppress uterine leiomyoma cell growth in vitro and in a tumor xenograft animal model [33,34]. Herein, we used EGCG as a positive control and, consistently, our results showed that treatment with epigallocatechin gallate (EGCG; 100 μM) inhibited cell growth in both cell types. In contrast, treatment of hUM cells with AHE-hex and ATE-ex at 200 μg/mL for 4 days reduced cell growth by approximately 30%. An approximately 30% reduction in hUM cell growth was similarly observed with AHE-ea treatment at 100 μg/mL, and an approximate 90% reduction with 200 μg/mL treatment was observed. The ATE-hex fraction did not affect hUM cell growth (Figure 4C).

The suppressive efficiency of the supernatant and sediment phase of AHE and ATE sub-fractions on hUL and hUM cells were evaluated. We found that treatment of hUL and hUM cells with each fraction exhibited similar results. hUL and hUM cells both treated with AHT-ea-S (100 μg/mL) for 4 days significantly reduced cell growth. However, both ATE-hex-L and ATE-hex-S exhibited lower efficiency on the suppression of cell growth (Figure 4D,E).

### 2.4. Identification of Potential Effective Compounds in the Ethyl Acetate Fraction of AHE (AHE-ea)

**Figure 5:**
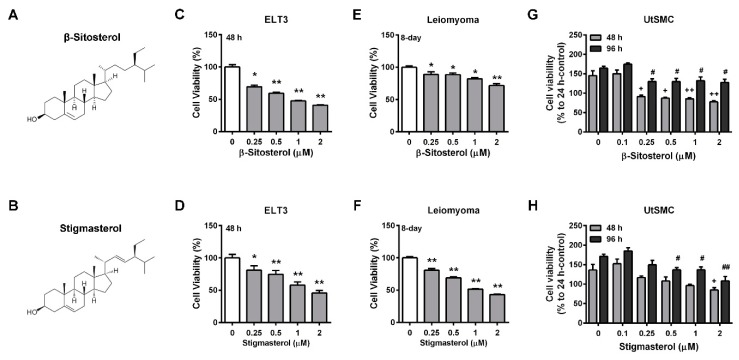
Effects of phytosterols on the viability of ELT3 and primary human uterine leiomyoma (hUL) cells as well as human UtSMC cells. The chemical structure of (**A**) β-sitosterol and (**B**) stigmasterol. Treatment with β-sitosterol (0.25, 0.5, 1, and 2 μM) and stigmasterol (0.25, 0.5, 1, and 2 μM) for 48 h in (**C**,**D**) ELT3 cells as well as for 8 days in (**E**,**F**) hUL cells. Human normal UtSMC cells treated with (**G**) β-sitosterol and (**H**) stigmasterol at various concentrations (0.1 0.25, 0.5, 1, and 2 μM) for 48 and 96 h. At the end of incubation, the cell viability was detected using the MTT assay. Data represent means ± SEM (*n* = 6 in ELT3 and hUL cells; *n* = 4 in UtSMC cells). * *p* < 0.05, ** *p* < 0.01 compared with the control group.

The phytosterols levels in AHE-ea fractions were further analyzed using HPLC analysis. The major components of the phytosterols are shown in Table 3. β-sitosterol and stigmasterol were contained in the AHE-ea, AHE-ea-L and AHE-ea-S fractions. Campesterol was also found in the AHE-ea-S fraction. Therefore, we further evaluated whether β-sitosterol and stigmasterol can inhibit uterine leiomyoma growth. The chemical structures of β-sitosterol and stigmasterol are illustrated in Figure 5A,B, respectively. The results showed that ELT3 cells treated with both β-sitosterol and stigmasterol for 48 h exhibited cell growth in a dose-dependent manner (Figure 5C,D). Treatment of hUL cells with β-sitosterol and stigmasterol for 8 days reduced cell growth. However, the efficiency of stigmasterol on anti-proliferation was better than that of β-sitosterol (Figure 5E,F). The effects of β-sitosterol and stigmasterol on the viability of human primary UtSMC cells were also assessed. The results showed that treatment of normal primary UtSMC cells with β-sitosterol reduced cell growth at concentrations of 0.25 to 2 μM for 48 and 96 h (Figure 5G); however, stigmasterol treatment exhibited lower inhibitory ability in normal primary UtSMC cells. UtSMC treated with stigmasterol at 2 μM for 48 h demonstrated decreased cell growth, whereas after treatment for 96 h, the concentration of stigmasterol from 0.5 to 2 μM also demonstrated anti-proliferation ability (Figure 5H).

### 2.5. Inhibition of Uterine Myometrial Growth by AHE-ea Fraction In Vivo

**Figure 6:**
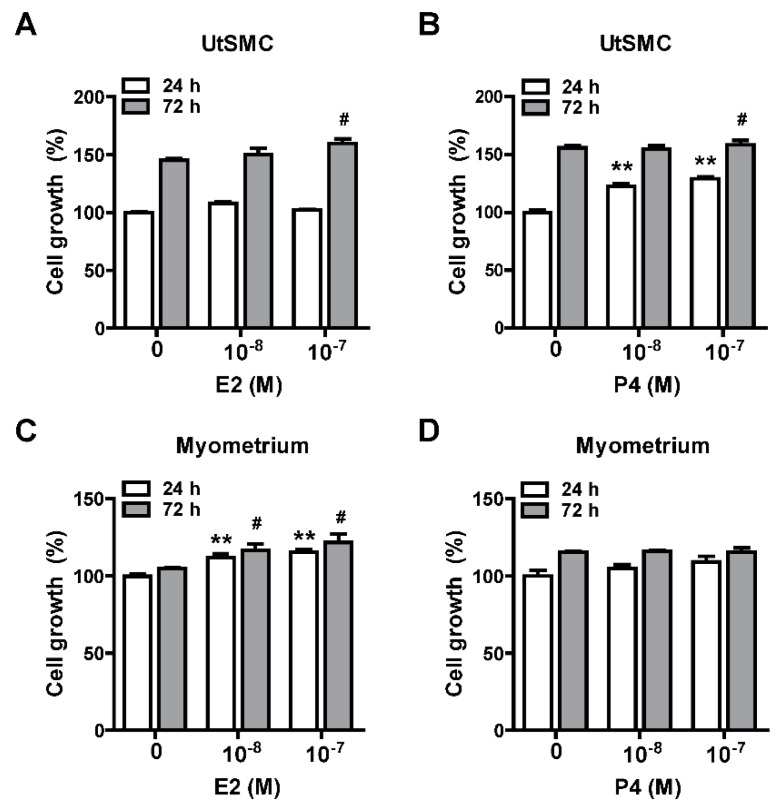
Effects of female sexual hormones on the growth of UtSMC and primary human uterine leiomyoma (hUM) cells. Treatment with estradiol (E2; 10^−8^ and 10^−7^ M) and progesterone (P4; 10^−8^ and 10^−7^ M) for 24 and 72 h in UtSMC (**A**,**B**) and human primary myometrial (hUM) cells (**C**,**D**). The cell viability was detected using the MTT assay. Data represent means ± SEM (*n* = 6). * *p* < 0.05, ** *p* < 0.01 compared with the control group.

**Figure 7:**
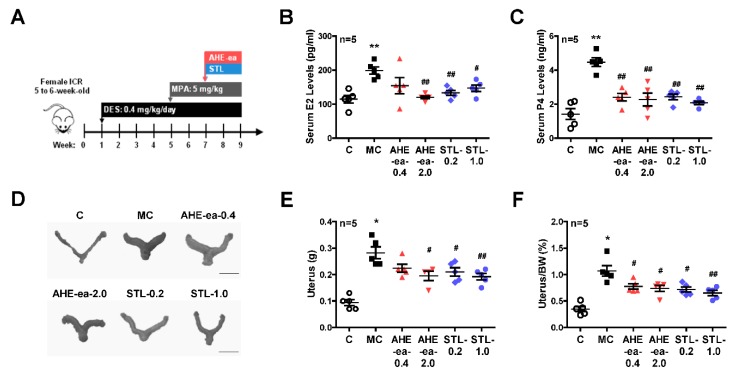
Effects of the ethyl acetate fraction of AHE on the DES/MPA-induced uterine leiomyoma in mice. (**A**) Diagram of the uterine leiomyoma mice model via DES and MPA induction. Female (5–6 weeks old) ICR mice were treated with DES (0.4 mg/kg) by oral gavage once daily for 4 weeks. After DES treatment for 4 weeks, mice were cotreated with DES and MPA (5 mg/kg) for an additional 4 weeks. At the 7th week of DES/MPA treatment, mice were treated with or without the ethyl acetate fraction of AHE (0.4 and 2 g/kg) and/or stigmasterol (0.2 and 1 mg/kg) by oral gavage once daily for 2 weeks. After sacrifice, the serum levels of (**B**) E2 and (**C**) P4 were measured using ELISA. The uteri were harvested and (**D**) photographed and weighted. (**E**) The weights of uteri were (**F**) normalized to the body weights. Data represent means ± SEM (*n* = 5). * *p* < 0.05, ** *p* < 0.01 compared with the control group. ^#^ *p <* 0.05, ^##^ *p <* 0.01 compared with the MC group. Scale bar = 1 cm. C, control. MC, model control. STL, stigmasterol.

**Figure 8:**
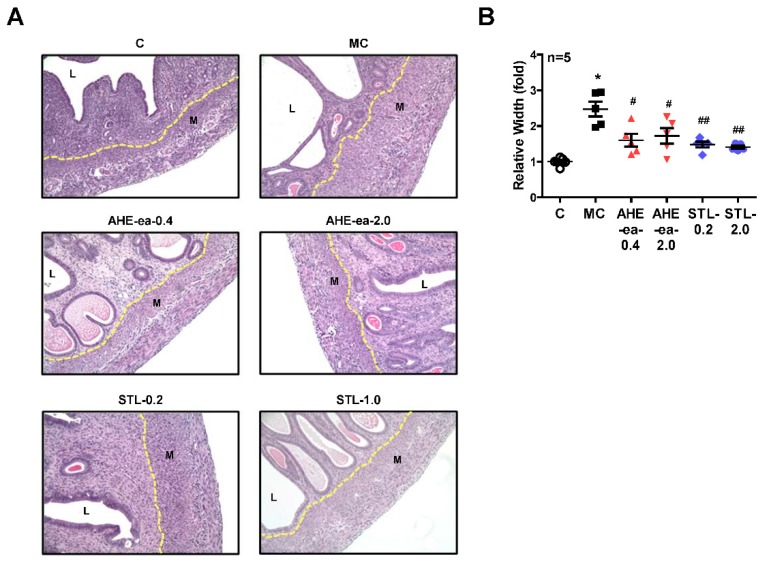
Effects of ethyl acetate fraction of AHE on uterine hyperplasia. After treatment with the ethyl acetate fraction of AHE or stigmasterol for 2 weeks in the DES/MPA-induced uterine hyperplasia mice model, uteri were harvested, formalin-fixed, and paraffin-embedded. (**A**) Four micrometer cross-sections of tissues were collected onto slides and then stained with hematoxylin and eosin (H&E). Magnification: 200×. (**B**) Quantitative analysis of the width of the myometrial area in a cross-section. Data represent means ± SEM (*n* = 5). * *p* < 0.05 compared with the control group. ^#^ *p* < 0.05, ^##^ *p* < 0.01 compared with the MC group. L, luminal space. M, myometrium. MC, model control. STL, stigmasterol.

Female sexual hormones have been reported to play a role in the enhancement of uterine leiomyoma growth, which is the critical step for further development of uterine leiomyoma [35,36]. Therefore, we first identified whether treatment with female sexual hormones, estradiol (E2) and progesterone (P4), induces UtSMC and hUM cell growth. The results showed that UtSMC cells treated with E2 at 10^−7^ M for 72 h increased cell growth (Figure 6A) and treatment with P4 for 24 and 72 h also increased cell growth (Figure 6B). In contrast, hUM cells treated with E2 at 10^−8^ and 10^−7^ M for 24 and 72 h, respectively, all displayed increased cell growth (Figure 6C). However, treatment with P4 did not alter cell growth (Figure 6D). These results suggested that female sexual hormones were involved in the regulation of uterine myometrium proliferation. Hence, we generated a mouse uterine myometrial hyperplasia model by treatment with diethylstilbestrol and medroxyprogesterone 17-acetate (DES and MPA), which are synthetic analogs of E2 and P4, respectively. The mouse in vivo model was created as shown in Figure 7A. The AHE-ea fraction and stigmasterol (STL) were treated by oral gavage, respectively, for 2 weeks. At the end of treatment, the serum E2 and P4 were measured and the weights of the uteri were recorded. The results showed that the serum levels of E2 and P4 increased in the model control (MC) group compared with the control group. Both serum E2 and P4 levels were reduced in AHE-ea and STL treatment groups, regardless of low or high dosage (Figure 7B,C). The morphology of uteri was photographed after scarification. The results showed that the weights of uterus’ significantly increased in the MC group. However, this increased phenotype was reduced in mice treated with AHE-ea and STL at both dosages (Figure 7D,E). Histopathological analyzes were performed on the uteri tissues. According to the H&E staining results, the uterine myometrial layer in the MC group increased compared with the control group. DES/MPA-induced uterine hyperplasia was also blocked by treatment with AHE-ea and STL alone at both dosages (Figure 8A,B).

## 3. Discussion

Uterine leiomyomas occur in approximately 25% of women during their reproductive life. However, of these, more than 25% of women with uterine leiomyomas show no symptoms and the condition may not be diagnosed. The severity of symptoms typically depends on size, number, and location of the fibroids [37,38]. The common symptoms of uterine leiomyomas include abnormal uterine bleeding, heavy and prolonged bleeding, painful menstruation, pelvic pain and pressure, dyspareunia, and reproductive dysfunction [2]. Functional impairment is caused by uterine leiomyomas that possibly reduce the quality of life for women. Although surgical and radiological therapies are often the first line treatment of these tumors, medical treatments are also considered as a therapeutic strategy for leiomyomas. Besides the synthetic chemical compounds, plant-based medicines can be selected for the treatment of tumors.

Previously, we reported that the combination of the hexane fraction of the adlay hull ethanolic extract (AHE-hex) and doxorubicin, which is widely used as a chemotherapeutic medicine, can decrease multidrug resistance and increase the synergistic effect on human uterine sarcoma cancer cells [31]. In the present study, we found that both ethanolic extracts of adlay hull (AHE) and adlay testa (ATE) demonstrated the ability to suppress rat uterine leiomyoma ELT3 cell growth. AHE and ATE and their supernatant and sediment fractions were further partitioned by mixing with n-hexane and ethyl acetate, respectively. All the sub-fractions at 400 μg/mL suppressed ELT3 and primary human uterine myometrial (UtSMC) cell growth, but the ethyl acetate fraction of AHE (AHE-ea) displayed this inhibitory effect at a lower concentration (200 μg/mL in UtSMC and 100 μg/mL in ELT3 cells). AHE and ATE were separated into supernatant and sediment fractions, then they were partitioned by n-hexane and ethyl acetate. We found that the AHE-ea-S fraction can suppress ELT3 and UtSMC cell growth. Consistently, primary human uterine leiomyoma (hUL) and normal uterine myometrial (hUM) cells treatment with AHE-ea and AHE-ea-S, respectively, significantly inhibited cell growth. These results suggested that AHE-ea can more efficiency suppress uterine leiomyoma growth and prevent normal uterine myometrium hyperplasia.

Plant-based medicines are regarded as potential therapeutic options for uterine leiomyoma. For instance, isoliquiritigenin (ISL), a natural chalcone flavonoid richly present in licorice and shallots, demonstrated an inhibitory effect on uterine leiomyoma growth by inducing the apoptotic pathway activation [39]. Resveratrol, which is widely present in red wine and grape skin, has been identified as having an anti-metastatic effect on malignant cancer cells [40]. We have also reported that resveratrol prevents the hyperplasia of leiomyoma and uterine smooth muscle cells [41]. In our previous study, we evaluated the anti-proliferation property of two classical compounds, phenolic compounds and flavonoids, from adlay on the growth rate of rat uterine leiomyoma ELT3 cells. We found that four pure compounds belonging to the flavonoids, including naringenin, quercetin, nobiletin, and eriodictyol, could suppress ELT3 cell growth. However, ELT3 treatment with 10 pure phenolic compounds did not affect cell growth [42]. In the present study, we found three phytosterols present in the AHE-ea, AHE-ea-S, and AHE-ea-L fractions. Campesterol was identified in the AHE-ea-s fraction alone, whereas β-sitosterol and stigmasterol were present in all three fractions. β-sitosterol and stigmasterol treatment produced a similar ELT3 cell growth suppression. Treatment with stigmasterol more effectively suppressed hUL cells compared with β-sitosterol. Treatment with stigmasterol caused less toxicity in normal in normal UtSMC cells compared to β-sitosterol. These results suggested that β-sitosterol and stigmasterol potentially contributed to the inhibitory effect of the AHE-ea fraction in terms of suppressing uterine leiomyoma growth.

Menstruating women have been reported to have a higher risk of uterine leiomyoma compared to postmenopausal women, implying that ovarian steroids, estrogen, and progesterone, are essential for uterine leiomyoma growth [5,35,43]. In the present study, our results showed that the proliferation of primary human uterine smooth muscle cells (UtSMC) and primary human uterine myometrial (hUM) cells increased with E2 and P4 treatment. In agreement with our findings, exogenous estrogen and progesterone exposure, such as estrogen-containing contraceptives and menopausal hormone therapy, have also been associated with an increased risk for uterine leiomyoma incidence [44,45,46,47]. In our animal study, we found that the weight of the uterus increased by co-treating mice with DES and MPA, which are the analogs of estrogen and progesterone, respectively. This increased effect was reduced by treatment with the AHE-ea fraction and stigmasterol alone. The DES- and MPA-increased serum levels of E2 and P4 were reduced by treatment with the AHE-ea fraction and stigmasterol alone. This result of the downregulation effect on E2 and P4 is consistent with our previous finding [48]. The DES/MPA-increased uterine myometrial layer was reduced by AHE-ea fraction and stigmasterol alone treatment. Stigmasterol was reported to suppress tumor growth via its anti-inflammatory activities [49] and/or through its anti-oxidant and anti-genotoxic properties [50]. E2 and P4 release from uterus involve the regulation of vascular endothelial growth factor (VEGF) expression [51]. VEGF expression has been reported to be higher in uterine leiomyoma compared with a uterine myometrial layer [52,53], suggesting that angiogenesis may be important for uterine leiomyoma development and growth. It has been reported that mice treated with stigmasterol inhibits cholangiocarcinoma growth through suppressing tumor angiogenesis by downregulation of tumor necrosis factor-alpha [49]. The expression of VEGFR2 was decreased by stigmasterol treatment, which in turn suppressed downstream signaling molecules’ activity [49]. Taken together, we suggested that the AHE-ea fraction and its potential compound, stigmasterol, can decrease the female-sexual-hormone-production-associated risk of uterine leiomyoma.

In conclusion, this study showed that the ethyl acetate fraction of AHE (AHE-ea) appears to effectively suppress the growth of rat uterine leiomyoma ELT3 cells and primary human uterine leiomyoma cells. The potential pure compounds of antitumor proliferation, stigmasterol and β-sitosterol, were identified from the AHE-ea fraction. Based on the results of this study, we propose a new insight that AHE-ea can be considered as a natural therapeutic candidate for uterine leiomyoma.

## 4. Materials and Methods

### 4.1. Preparation of Ethanolic Extracts from Each Part of Adlay Seed

The Taichung Shuenyu No.4 (TSC4) species of adlay (*Coxi lachrymal-jobi* L.var. *ma-yuen* Stapf.; Taichung, Taiwan) was used in this study. A voucher specimen (no. 19789) has been deposited at the Herbarium of the Institute of Botany, Academia Sinica (Taipei, Taiwan). Adlay seeds were air-dried and separated into four parts: adlay hull, adlay testa, adlay bran, and polished adlay. These components were blended into a powder form and sieved through a 20-mesh sieve (aperture, 0.94 mm) [54].

The ethanolic extraction process was modified from our previous reports [31,42]. The flowchart of the ethanolic extraction process is illustrated in Figure 1A. Each powder part (100 g) from adlay seed was extracted with 10 times (*w*/*v*) the volume of 80% ethanol and placed at room temperature for 24 h, and then these supernatants were filtered and collected. The sediments were extracted with 10 times (*w*/*v*) the volume of 80% ethanol and placed at room temperature for 24 h. The ethanolic extraction step was repeated three times. After the third ethanolic extraction, all the supernatants were pooled and then concentrated to dryness under a vacuum to produce a dried powder from ethanolic extracts. The ethanolic extracts from these four parts of the adlay seed were named as follows: the ethanolic extract of adlay hull (AHE), the ethanolic extract of adlay testa (ATE), the ethanolic extract of adlay bran (ABE), and the ethanolic extract of polished adlay (PAE).

### 4.2. Preparation of Various Fractions from Ethanolic Extracts of Adlay Hull (AHE) and Testa (ATE).

To prepare different partitioned fractions, AHE and ATE powders were dissolved in 10 times (*w*/*v*) the distilled water and then vigorously mixed with an equal volume of n-hexane for 10 min. The mixtures were rested until two layers of solvent could be clearly observed. The hexane layers were collected and concentrated under vacuum to obtain the dried products that were labeled the hexane fraction of AHE (AHE-hex) and ATE (ATE-hex). Subsequently, the aqueous layers were concentrated to remove the residual hexane in this layer. Then, distilled water was added to the aqueous layers to fill it to the original volume before partition. These aqueous layers were vigorously mixed with 15 times (*w*/*v*) the volume of ethyl acetate for 10 min. Similarly, the mixtures were rested until two layers of solvent could be clearly observed. The ethyl acetate layers were collected and concentrated under a vacuum to obtain the dried products that were labeled the ethyl acetate fraction of AHE (AHE-ea) and ATE (ATE-ea). The process flowchart is illustrated in Figure 2A.

We placed the AHE and ATE at room temperature for 48 h, then subsequently separated the supernatants and sediments by centrifugation. The samples were concentrated under a vacuum, and the dried powders from supernatant and sediment of AHE and ATE were harvested. The supernatant part of AHE and ATE were named AHE-L and ATE-L, respectively; the sediment parts of AHE and ATE were named AHE-S and ATE-S, respectively. The hexane- and ethyl acetate-fractions were partitioned from AHE-L, AHE-S, ATE-L, and ATE-S. The flowchart is illustrated in Figure 3A. The process was performed similarly to that described above. Finally, the dried products were called the hexane fractions of supernatant of AHE or ATE (AHE-hex-L and ATE-hex-L, respectively), the hexane fractions of sediment of AHE or ATE (AHE-hex-S and ATE-hex-S, respectively), ethyl acetate fractions of supernatant of AHE or ATE (AHE-ea-L; ATE-ea-L), and ethyl acetate fractions of sediment of AHE or ATE (AHE-ea-S and ATE-ea-S, respectively). All extract powders were dissolved in DMSO to obtain 200 mg/mL stock concentration for all in vitro experiments in this study.

### 4.3. Cell Lines and Culture Conditions

The Eker rat-derived uterine leiomyoma (ELT3) cell line and primary human uterine smooth muscle (UtSMC) cells were kindly provided by Dr. Lin-Hung Wei (Department of Oncology, National Taiwan University Hospital, Taipei, Taiwan). Both ELT3 and UtSMC were maintained in Dulbecco’s Modified Eagle Medium/Ham’s F-12 Medium 1:1 (DMEM/F12, CAISSON Labs, Smithfield, UT, USA) supplemented with 10% fetal bovine serum (FBS; Biological Industries, Cromwell, CT, USA), 100 units/mL penicillin, 100 μg/mL streptomycin, sodium bicarbonate (2.438 g/L), and HEPES (5.986 g/L) in a humidified incubator (37 °C, 5% CO_2_).

### 4.4. Preparation of Primary Human Uterine Leiomyoma (hUL) Cells and Human Uterine Myometrial (hUM) Cells

Primary human uterine leiomyoma and its surrounding normal myometrial tissue specimens were collected from 30–40-year-old women (*n* = 6) undergoing myomectomy at the Department of Oncology, National Taiwan University Hospital (Taipei, Taiwan). In this study, all human tissue specimens were approved by the Institutional Review Board and Ethics Committee of National Taiwan University Hospital (Permit Number: 201210072RIC).

The purification process of human primary uterine leiomyoma (hUL) cells and human primary uterine myometrial (hUM) cells was performed as described previously [42]. This process was illustrated as Figure 4A. Briefly, we raised the tissues with glucose–potassium–sodium–phosphate (GKNP) solution containing 1% antibiotics and then tissues were cut into small fractions and incubated in the DMEM/F-12 medium with 10% FBS and 0.2% collagenase (Sigma-Aldrich, Louis, MO, USA) at 37 °C water bath with gentle agitation for 3–4 h. The mixture was filtered using 70 μm sterilized mesh and the suspension was collected and centrifugation was performed at 300× *g* for 5 min to remove the collagenase-contacting medium. The cell pellet was resuspended with DMEM/F-12 culture medium supplemented with 10% FBS and then centrifuged again. Finally, cells were plated into a plastic culture dish and incubated in a humidified incubator (37 °C) with 5% CO_2_. To identify the purity of the leiomyoma and normal myometrial cells from this procedure, we monitored the markers of the expression level of the myometrial marker protein, such as alpha-smooth muscle action, using immunoblotting analysis (data not shown). Cells from passages 2–7 were used in this study.

### 4.5. Cell Survival Assay

MTT (3-[4,5-dimethyl-2-thiazolyl]-2,5-diphenyl-2H-tetrazolium bromide, Sigma-Aldrich) assay was performed to evaluate the effects of various fractions of AHE and ATE on the growth rate of ELT3, UtSMC, hUL, and hUM cells. Cells (2500 cells/well for ELT3 and UtSMC cells and 2000 cells/well for hUL and hUM cells) were seeded in 96-well microplates with 100 μL/well culture medium. After cell attachment on the bottom of the well, cells were then treated with the challenge medium with various fractions derived from AHE and ATE. At the end of incubation, the media were removed and replaced by serum-free culture medium with 0.5 mg/mL MTT, and then cells were incubated for an additional 4 h. Subsequently, the media were removed and crystal formazan was dissolved using 100 μL/well DMSO (Sigma-Aldrich). The optical density was measured using a microplate reader (BioTek, Winooski, VT, USA) at 570 nm and 630 nm as the reference wavelength.

### 4.6. High-Performance Liquid Chromatography (HPLC) Analysis

The process we used was followed and partially modified by a previous study [31]. The separation of the experimental samples was conducted using the ACQUITY Ultra Performance LC system (Waters, Milford, MA, USA) with a detector MICROMASS Quattro Premier XE (Waters) that was equipped with a Waters BEH RP18 (2.1 mm x 100 mm, 1.7μm) column (Waters). The 20 μL sample was injected into the column. Two solutions were used in mobile system, solution A: 0.1% formic acid/H_2_O (98:2, *v*/*v*) and solution B: 0.1% formic acid/methanol (98:2, *v/v*). The mobile phase was a mixture solution of A/B (10:90 (%) until 5 min, 5:95 (%) until 10 min, 0:100 until 15 min, then 10:90 (%) until 10 min) with a flow rate of 0.2 mL/min. The peak areas were recorded using Chromatography Data System software (Scientific Information Service Corporation Inc., Taipei, Taiwan).

### 4.7. Animals

Six-week-old female Institute of Cancer Research (ICR) [CD-1] mice were purchased from BioLASCO Taiwan Corporation (Taipei, Taiwan) and were housed with a 12 h light/dark artificial illumination cycle (0800–2000) in a temperature-controlled room (22 ± 2 °C). Food and water were provided ad libitum. All animal experimental procedures in this study were in accordance with the approved guidelines and regulations and were approved by the Institutional Animal Care and Used Committee (IACUC), Taipei Medical University.(LAC-2014-0011)

The mouse uterine hyperplasia animal model generation process was illustrated in Figure 7A. After adaptation for 1 week, mice were randomly divided into six groups as follows. The control group mice were treated with corn oil by oral gavage once daily until the end of the experiment. The model control (MC) group was generated by oral gavage with diethylstilbestrol (DES, 0.4 mg/kg; Sigma-Aldrich) once daily for 4 weeks and consequently with co-treatment with DES and medroxyprogesterone 17-acetate (MPA, 5 mg/kg; Sigma-Aldrich) once daily for an additional 4 weeks. At the 7th week of DES/MPA treatment, two groups of mice were treated with the ethyl acetate fraction of AHE (AHE-ea) at 0.4 and 2.0 g/kg by oral gavage once daily for 2 weeks, referred to as AHE-ea-0.4 and AHE-ea-2.0, respectively. During the 7th week of DES/MPA treatment, two groups of mice were treated with stigmasterol (STL) at 0.2 and 1 mg/kg by oral gavage once daily for 2 weeks, referred to as STL-0.2 and STL-1.0, respectively, that acted as a positive control in this study.

All mice were sacrificed through decapitation. Blood samples were collected from the heart and serums were isolated to further analyze the levels of E2 and P4. Uteri were quickly isolated and photographed and weighted.

### 4.8. Enzyme-Linked Immunosorbent Assay (ELISA) of Serum E2 and P4

The concentrations of serum E2 and P4 were measured using the E2 ELISA kit (Cayman Chemical, Ann Arbor, MI, USA) and P4 ELISA kit (Cayman Chemical), respectively. All procedures were performed according to the manufacturer’s protocols. The absorbance values of these two hormone kits were measured using a microplate reader (BioTek) at 420 nm.

### 4.9. H&E Staining

Uterine tissues were fixed in 10% formalin for 24 h and then dehydrated using serial percentage alcohols and embedded in paraffin. Four-micrometer cross sections were collected onto slides. Tissue sections were stained with hematoxylin and eosin by the National Laboratory Animal Center (Taipei, Taiwan). Images were photographed at 200x magnifications using a microscope.

### 4.10. Statistical Analysis

All data are expressed as mean ± standard error of the mean (SEM). Data evaluated the difference among all groups using one-way ANOVA. Student’s unpaired *t*-test was used for a comparison between the two groups. Statistical analysis was performed using Prism version 5.0 software (GraphPad, San Diego, CA, USA). At a *p* value < 0.05, significance was statistically accepted.

## Figures and Tables

**Table 1:** Yield of sub-fractions from AHE and ATE.

Compound	Weight (g)	Yield (%)
AHE-hex	26.7	0.19
AHE-ea	18.1	0.12
ATE-hex	90.9	1.31
ATE-ea	50.6	0.73

**Table 2:** Yield of sub-fractions from supernatant and sediment of AHT and ATE.

Compound	Weight (g)	Yield (%)
AHE-hex-L	7.5	0.04
AHE-ea-L	18.9	0.09
AHE-hex-S	23.9	0.12
AHE-ea-S	10.2	0.05
ATE-hex-L	19.5	0.24
ATE-ea-L	9.9	0.12
ATE-hex-S	88.7	1.11
ATE-ea-S	34.3	0.43

Yield: sub-fraction weight/adlay hull or adlay testa dry weight.

**Table 3:** Phytosterols content from different sub-fraction of AHE.

Sample	β-Sitosterol	Stigmasterol	Campesterol
AHE-ea	522.80	216.24	Trace
AHE-ea-L	21.61	23.88	Trace
AHE-ea-S	2243.29	1417.84	588.74

unit: μg/g dry weight of adlay hull.

## Abbreviations

ABE, ethanolic extract of adlay bran; AHE, ethanolic extract of adlay hull; AHE-ea, ethyl acetate fraction of adlay hull ethanolic extract; AHE-ea-L, ethyl acetate fractions of supernatant of adlay hull ethanolic extract; AHE-ea-S, ethyl acetate fractions of sediment of adlay hull ethanolic extract; AHE-hex, hexane fraction of adlay hull ethanolic extract; AHE-hex-L, hexane fractions of supernatant of adlay hull ethanolic extract; AHE-hex-S, hexane fractions of sediment of adlay hull ethanolic extract; ATE, ethanolic extract of adlay testa; ATE-ea, ethyl acetate fraction of adlay testa ethanolic extract; ATE-ea-L, ethyl acetate fractions of supernatant of adlay testa ethanolic extract; ATE-ea-S, ethyl acetate fractions of sediment of adlay testa ethanolic extract; ATE-hex, hexane fraction of adlay testa ethanolic extract; ATE-hex-L, hexane fractions of supernatant of adlay testa ethanolic extract; ATE-hex-S, hexane fractions of sediment of adlay testa ethanolic extract; COX-2, cyclooxygenase-2; DES, diethylstilbestrol; E2, estradiol; EG, epigallocatechin gallate (EGCG); ELISA, Enzyme linked immunosorbent assay; ELT3, Eker rat-derived uterine leiomyoma cells; GKNP, glucose-potassium-sodium-phosphate buffer; HPLC, High-performance liquid chromatography; hUL, primary human uterine leiomyoma cells; hUM, normal uterine myometrial (hUM) cells; ICR, Institute of Cancer Research; ISL, isoliquiritigenin; KLTi, Kanglaite injection; MC, model control; P4, progesterone.
